# The Trace Element Selenium Is Important for Redox Signaling in Phorbol Ester-Differentiated THP-1 Macrophages

**DOI:** 10.3390/ijms222011060

**Published:** 2021-10-14

**Authors:** Theresa Wolfram, Leonie M. Weidenbach, Johanna Adolf, Maria Schwarz, Patrick Schädel, André Gollowitzer, Oliver Werz, Andreas Koeberle, Anna P. Kipp, Solveigh C. Koeberle

**Affiliations:** 1 Department of Nutritional Physiology, Institute of Nutritional Sciences, Friedrich Schiller University Jena, 07743 Jena, Germany; theresa.wolfram@uni-jena.de (T.W.); leonie.weidenbach@uni-jena.de (L.M.W.); johanna232@gmx.net (J.A.); schwarz.maria@uni-jena.de (M.S.); 2 Department of Pharmaceutical/Medicinal Chemistry, Institute of Pharmacy, University of Jena, 07743 Jena, Germany; patrick.schaedel@uni-jena.de (P.S.); oliver.werz@uni-jena.de (O.W.); 3 Michael Popp Institute and Center for Molecular Biosciences Innsbruck (CMBI), University of Innsbruck, 6020 Innsbruck, Austria; andre.gollowitzer@uibk.ac.at (A.G.); andreas.koeberle@uibk.ac.at (A.K.); 4 Institute of Pharmacy/Pharmacognosy and Center for Molecular Biosciences Innsbruck (CMBI), University of Innsbruck, Innrain 80-82, 6020 Innsbruck, Austria

**Keywords:** selenium, selenoprotein, macrophage, differentiation, inflammation, redox signaling, NRF2, NF-κB, lipid mediators

## Abstract

Physiological selenium (Se) levels counteract excessive inflammation, with selenoproteins shaping the immunoregulatory cytokine and lipid mediator profile. How exactly differentiation of monocytes into macrophages influences the expression of the selenoproteome in concert with the Se supply remains obscure. THP-1 monocytes were differentiated with phorbol 12-myristate 13-acetate (PMA) into macrophages and (i) the expression of selenoproteins, (ii) differentiation markers, (iii) the activity of NF-κB and NRF2, as well as (iv) lipid mediator profiles were analyzed. Se and differentiation affected the expression of selenoproteins in a heterogeneous manner. GPX4 expression was substantially decreased during differentiation, whereas GPX1 was not affected. Moreover, Se increased the expression of selenoproteins H and F, which was further enhanced by differentiation for selenoprotein F and diminished for selenoprotein H. Notably, LPS-induced expression of NF-κB target genes was facilitated by Se, as was the release of COX- and LOX-derived lipid mediators and substrates required for lipid mediator biosynthesis. This included TXB_2_, TXB_3_, 15-HETE, and 12-HEPE, as well as arachidonic acid (AA), eicosapentaenoic acid (EPA), and docosahexaenoic acid (DHA). Our results indicate that Se enables macrophages to accurately adjust redox-dependent signaling and thereby modulate downstream lipid mediator profiles.

## 1. Introduction

Selenium (Se) is an essential trace element that is important for human health. It is required for various physiological processes including immune function and mammalian development, as well as thyroid hormone metabolism, and Se deficiency has been implicated in various pathologies including inflammation, heart disease, and cancer [1,2]. The biological effects of Se are mainly mediated by selenoproteins, which contain the amino acid selenocysteine, the Se-containing analogue of cysteine, in their polypeptide chain [2]. Most commonly, selenocysteine is part of the catalytic center and provides the selenoproteins with unique chemical activities due to the superior reactivity of selenocysteine. Accordingly, many selenoproteins regulate redox-dependent processes [3] and adequate levels of Se and selenoproteins have been reported to be critical for proliferation, differentiation, and inflammatory processes [4]. The impact of individual selenoproteins is thereby dependent on the cell type and differentiation state. Hence, expression levels of selenoproteins largely differ between undifferentiated and differentiated cells, which suggests that distinct selenoproteins inherit specific functions depending on the differentiation state. Knock-down of selenoprotein (SELENO)H, for example, triggers proliferation of undifferentiated human HT-29 colorectal carcinoma cells and decreases their differentiation, while SELENOH is significantly downregulated in differentiated HT-29 cells [5]. In contrast, thioredoxin reductases (TXNRDs) are upregulated in differentiated adiopocytes and inhibit the differentiation process [6,7]. Moreover, various other selenoproteins, such as glutathione peroxidases (GPXs), SELENOO, and SELENOK, have been shown to be implicated in proliferation and differentiation of distinct cell types [4,8,9].

Macrophages, which are derived from circulating monocytes, are important cells of the innate immune system [10]. Depending on the microenvironmental signals, macrophages give rise to a variety of different tissue-resident-activated macrophage sub-populations, including pro-inflammatory M1- or anti-inflammatory M2-type macrophages [11,12]. Two redox-regulated transcription factors, nuclear factor κ-light-chain-enhancer (NF-κB) and nuclear factor erythroid 2-related factor 2 (NRF2), are key regulators of inflammation and oxidative stress-induced cellular responses in macrophages. These play pivotal roles in host defense and inflammatory processes but are also important for tissue homeostasis and repair [13,14]. While NF-κB mainly triggers gene expression of inflammatory proteins like cyclooxygenase (COX)2 and tumor necrosis factor (TNF)α, thereby enhancing oxidative stress, NRF2 activates the antioxidant response, which includes the upregulation of TXNRD1, GPX4, superoxide dismutase (SOD)1, heme oxygenase (HMOX)1, and catalase (CAT), as well as proteins involved in glutathione (GSH) homeostasis, including solute carrier family 7 member 11 (SLC7A11), glutamate-cysteine ligase catalytic subunit (GCLC), and glutamate-cysteine ligase modifier subunit (GCLM). The two pathways are cross-linked by a complex network, thereby allowing fine-tuned inflammatory responses and preventing exacerbated oxidative stress and inflammation [15]. Various studies suggest that adequate Se levels limit excessive inflammation [2], and genetic inhibition studies identified specific functions of individual selenoproteins in the macrophage maturation process (GPX1 and SELENOP), as well as in the limitation of the oxidative burst (SELENOK) [16].

In mammals, the hierarchy of selenoproteins ensures that indispensable selenoproteins are expressed at the expense of less important ones when the Se supply is limited [17]. A second hierarchical principle ensures the transport of Se to privileged tissues, e.g., the brain. The expression pattern of selenoproteins is highly dynamic, tightly regulated, and fine-tuned depending on the developmental stage, physiological conditions, and the availability of Se [1,18,19].

Given that adequate expression of selenoproteins (i) depends on Se supply, (ii) varies widely between different tissue types, (iii) can be altered by the differentiation process, and (iv) is required for an appropriate inflammatory response, this raises the questions of how the Se status affects monocyte/macrophage function and how selenoprotein expression levels are regulated in macrophages during the differentiation process. Human THP-1 monocytes have been suggested to be a valuable model for macrophage differentiation because phorbol 12-myristate 13-acetate (PMA)-differentiated THP-1 macrophages resemble the native monocyte-derived macrophages with regard to (i) morphology, (ii) expression of membrane antigens and receptors, (iii) the release of secretory factors, and (iv) transient induction of proto-oncogenes [20]. We established a THP-1 model to investigate the effect of Se on (i) cell proliferation and differentiation of monocytes and (ii) the expression of selenoproteins in undifferentiated monocytes as compared to differentiated macrophages, as well as (iii) the inflammatory response upon lipopolysaccharide (LPS) stimulation. Interestingly, Se treatment neither significantly affected the proliferation of human THP-1 monocytes nor the differentiation process itself. However, expression of almost all selenoproteins was modulated by the differentiation state of THP-1 cells. Adequate levels of Se were moreover important for the LPS-induced inflammatory response, resulting in an upregulation of NF-κB target gene expression as well as increased biosynthesis of lipid mediators (LMs). In conclusion, the tight regulation of the selenoproteome by both Se and differentiation processes together with the dependency of the inflammatory response on Se levels strongly suggest that an adequate Se status is indispensable for proper functioning of macrophages.

## 2. Results

### 2.1. Se Does Not Affect Differentiation of THP-1 Cells into Macrophages

To investigate the effect of Se on the differentiation process of human THP-1 leukemic monocytes into macrophages, we initially determined the Se concentration and incubation time required for adequate expression of Se-sensitive selenoproteins. Concentration-dependent studies with 0–500 nM sodium selenite for 72 h confirmed a significant upregulation of the protein expression levels of the Se-sensitive selenoproteins GPX1 and SELENOH (Figure S1a,b), while expression of TXNRD1 was not affected (Figure S1c). Moreover, protein expression of GPX1, SELENOH, and TXNRD1 was time-dependently upregulated, reaching a maximum after 72 h (Figure S1d–f). Cell number was not significantly affected up to 72 h by 50 nM Se treatment (Figure S1g). Pre-treatment with 50 nM Se for 72 h was therefore used for subsequent experiments.

**Figure 1:**
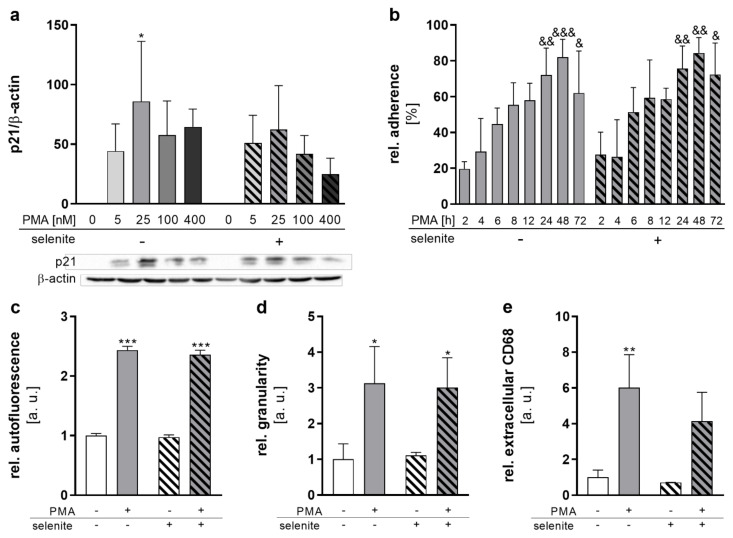
Selenium does not affect differentiation. THP-1 monocytes were pre-treated for 72 h with or without 50 nM sodium selenite (selenite) and differentiated into macrophages by 25 nM PMA or the indicated concentration with or without 50 nM selenite for the indicated time points (**b**) or for 48 h (**a**,**c**–**e**). (**a**) Protein expression of p21 was normalized to β-actin. -PMA/-selenite samples were set to 1. A representative blot is shown. (**b**) The relative adherence was calculated as (number of adherent cells/total number of cells) × 100. (**c**–**e**) The autofluorescence (**c**), intracellular granularity (**d**), and extracellular CD68 (**e**) were determined using flow cytometry. Data are given as means + SD (n = 3–4). Two-way ANOVA with Bonferroni’s post-test. Significant outliers were determined by Grubbs’ test (α = 0.05). * *p* < 0.05, ** *p* < 0.01, *** *p* < 0.001 vs. cells without PMA (PMA effect); ^&^ *p* < 0.05, ^&&^ *p* < 0.01, ^&&&^ *p* < 0.001 vs. 2 h PMA.

THP-1 differentiation into macrophages can be induced by PMA and is associated with increased cell size and adherence [21,22]. Concentration–response studies (0–100 nM PMA) showed that treatment with low (5 nM) PMA concentrations for 48 h was not sufficient to induce complete differentiation, as indicated by loosely attached cells with monocytic morphology (Figure S1h). Incubation with 25 nM for 48 h induced a pronounced adherence of the cells, with significantly increased cell sizes (Figure S1h). Higher concentrations of PMA (100 nM) did not further increase adherence or cell size. Recent studies indicate that high PMA concentrations induce a rather pro-inflammatory type of macrophage which is less responsive towards stimuli [22,23]. Hence, 25 nM of PMA was used for differentiation. In addition, the cell cycle inhibitor p21WAF1/Cip1 was induced by PMA reaching a maximum at 25 nM PMA (Figure 1a). This effect was independent of Se treatment. Time-dependent studies (2–72 h) with 25 nM PMA confirmed a progressive increase in relative cell adherence, reaching a maximum after 48 h (Figure 1b). To further explore the influence of Se on the PMA-induced THP-1 differentiation process, cellular autofluorescence and granularity, as well as extracellular CD68 levels, were analyzed. All three differentiation markers increased with PMA treatment, whereas Se did not significantly affect differentiation (Figure 1c–e). In summary, differentiation of monocytes into macrophages was not significantly affected by the Se status of the cells.

### 2.2. PMA-Induced Differentiation of Monocytes to Macrophages Alters the Selenoprotein Expression Profile

**Figure 2:**
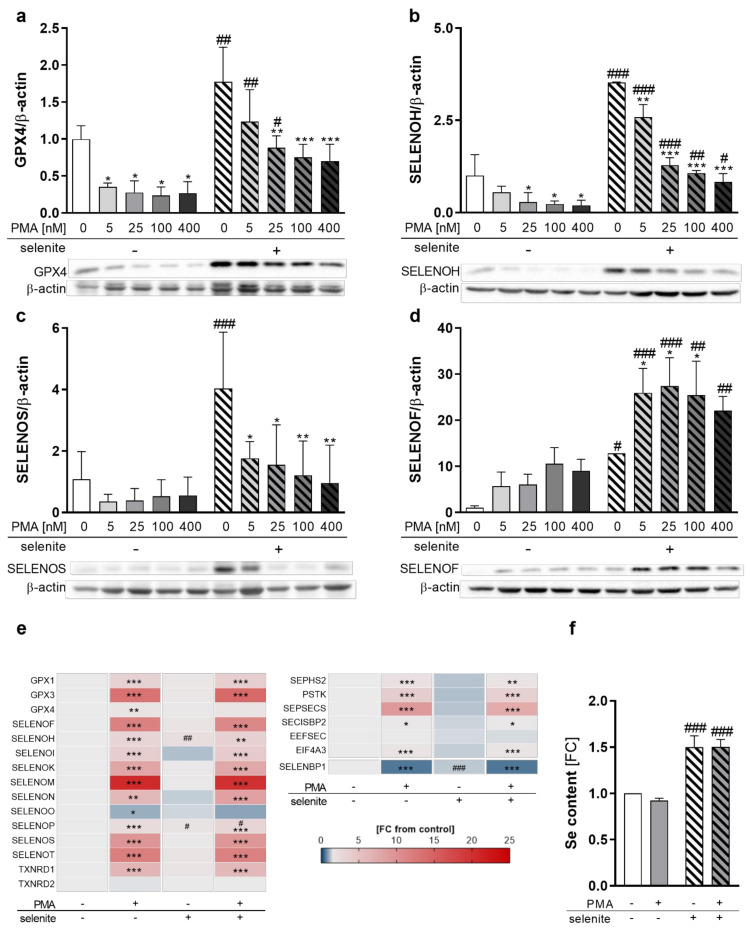
Expression of selenoproteins as well as proteins involved in selenoprotein biosynthesis during differentiation. THP-1 monocytes were pre-treated for 72 h with or without 50 nM sodium selenite (selenite) and differentiated into macrophages using 5–400 nM PMA (**a**–**d**) or 25 nM PMA (**e**,**f**) with or without 50 nM sodium selenite treatment for 48 h. (**a**–**d**) Protein expressions of GPX4 (**a**), SELENOH (**b**), SELENOS (**c**), and SELENOF (**d**) were normalized to β-actin. -PMA/-selenite samples were set to 1. Representative blots are shown. (**e**) Heatmap of mRNA expression of different selenoproteins and proteins involved in the biosynthesis of selenoproteins analyzed by qRT-PCR. The color scale indicates the mean difference as fold change (FC) of target genes vs. -PMA/-selenite (FC = 1). (**f**) Se content of the cells. -PMA/-selenite samples were set to 1. Data are given as means + SD (n = 3–4). Two-way ANOVA with Bonferroni’s post-test. Significant outliers were determined by Grubbs´ test (α = 0.05). * *p* < 0.05, ** *p* < 0.01, *** *p* < 0.001 vs. cells without PMA (PMA effect); ^#^ *p* < 0.05, ^##^ *p* < 0.01, ^###^ *p* < 0.001 vs. cells without selenite (Se effect).

To investigate whether selenoprotein expression is affected by differentiation in combination with varying Se supply, we analyzed the protein levels of selenoproteins under Se-deficient, as well as Se-adequate, conditions at increasing PMA concentrations. As expected, selenoprotein expression was upregulated in Se-treated cells, as shown for GPX4, SELENOH, SELENOS, and SELENOF (Figure 2a–d). Most pronounced Se-dependent effects were obtained for SELENOF in undifferentiated THP-1 monocytes with a 12-fold upregulation, followed by SELENOS and SELENOH. GPX4 expression was only moderately affected, being upregulated by 1.8-fold in Se-treated cells. In contrast, PMA substantially reduced expression levels of the selenoproteins SELENOH, SELENOS, and GPX4 in both Se-treated as well as untreated cells (Figure 2a–c). However, the protein expression of SELENOF increased upon PMA-induced differentiation (Figure 2d), while other selenoproteins, such as GPX1, SELENOO, TXNRD1, and TXNRD2, were almost unaffected by PMA-induced differentiation (Figure S2a–d). Accordingly, total activities of the TXNRD and GPX selenoprotein families were also not modulated by PMA (Figure S2e,f).

As changes in selenoprotein expression upon PMA treatment can be regulated at either the transcriptional or translational levels, we investigated mRNA levels of the selenoproteins in untreated and PMA-differentiated THP-1 cells. Interestingly, mRNA levels were not related to protein expression. The only selenoprotein which was downregulated by PMA on mRNA level was SELENOO (Figure 2e). Significant upregulation of mRNA levels was observed for all other selenoproteins investigated, with SELENOM showing the strongest effect with a 20-fold increase in macrophages as compared to monocytes (Figure 2e). Moreover, mRNA levels of Se-binding protein 1 (SELENBP1), a non-selenocysteine-containing protein that covalently binds Se [24,25], were downregulated by PMA (Figure 2e), which was also observed upon differentiation of other cell types [26,27].

Based on this discrepancy between mRNA and protein expression of selenoproteins, the mRNA levels of proteins involved in selenoprotein synthesis were analyzed. While expression of genes responsible for the biosynthesis of selenocysteine at the t-RNA^[Ser]Sec^ (e.g., selenophosphate synthetase 2 (SEPHS2), phosphoseryl-tRNA kinase (PSTK), sep (o-phosphoserine) tRNA:sec (selenocysteine) tRNA synthase (SEPSECS)) were significantly upregulated, mRNA expression of proteins involved in selenoprotein translation (SECIS binding protein 2 (SECISBP2) and eukaryotic elongation factor, selenocysteine-tRNA specific (EEFSEC)) were less affected by PMA treatment (Figure 2e). Interestingly, eukaryotic translation initiation factor 4A3 (EIF4A3), which inhibits the biosynthesis of Se-sensitive selenoproteins, was significantly upregulated by PMA (Figure 2e). Moreover, cellular Se levels were independent from the differentiation state and upregulated by Se treatment by 1.5-fold in undifferentiated and differentiated cells, which excludes the possibility that Se availability limited protein expression of selenoproteins in PMA-treated cells (Figure 2f). In conclusion, PMA-induced differentiation downregulated selenoprotein expression of GPX4, SELENOH, and SELENOS but upregulated SELENOF, indicating differentiation-induced shifts of the selenoproteome.

### 2.3. PMA and Se Treatment Modulate the Cellular Redox Status and the Activity of the Redox-Sensitive Transcription Factors NRF2 and NF-κB

**Figure 3:**
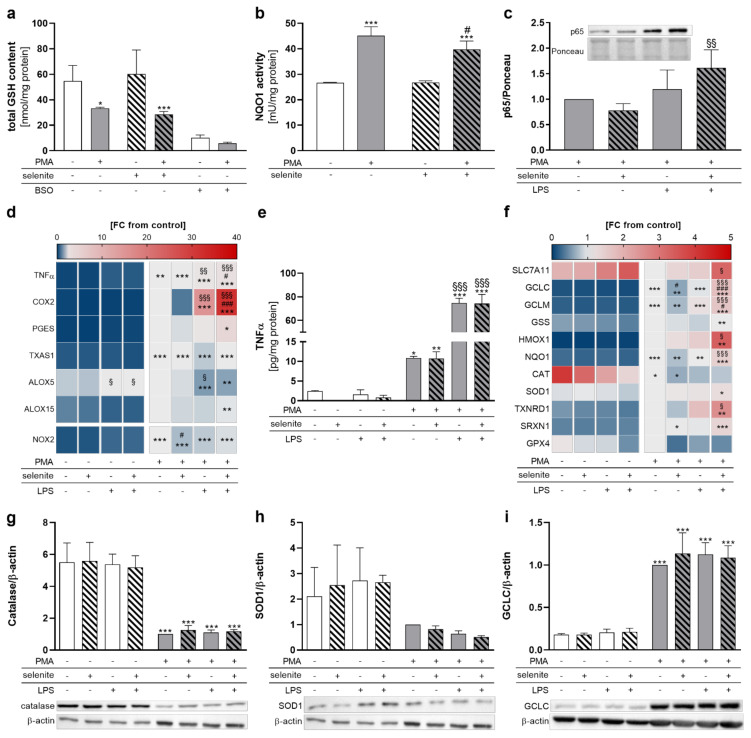
Redox-dependent regulation of NF-κB and NRF2 target gene transcription in THP-1 monocytes and macrophages. THP-1 monocytes were pre-treated with or without 50 nM sodium selenite (selenite) for 72 h and differentiated into macrophages with 25 nM PMA with or without 50 nM selenite treatment for 48 h. Cells were stimulated with 1 µg/mL lipopolysaccharide (LPS) (**c**–**i**) for 1 h (**c**), 6 h (**d**,**f**), or 24 h (**e**,**g**–**i**). (**a**) Total GSH content. The GSH synthesis inhibitor buthionine-sulfoximine (0.25 mM, BSO) was used as positive control. (**b**) NQO1 activity. (**c**) The protein expression of nuclear p65 was normalized to Ponceau staining. The -selenite/-LPS sample was set to 1. (**d**) Heatmap of mRNA expression of NF-κB target genes and enzymes involved in the LM biosynthesis investigated by qRT-PCR. mRNA expression of genes is given as fold change (FC) vs. +PMA/-selenite/-LPS (FC = 1). The color scale indicates the mean difference as the FC of target genes vs. +PMA/-selenite/-LPS (FC = 1). (**e**) TNFα release was analyzed by ELISA. (**f**) Heatmap of mRNA expression of NRF2 target genes investigated by qRT-PCR. mRNA expression of genes is given as fold change (FC) vs. +PMA/-selenite/-LPS (FC = 1). The color scale indicates the mean difference as the FC of target genes vs. +PMA/-selenite/-LPS (FC = 1). (**g**–**i**) The protein expressions of CAT (**g**), SOD1 (**h**), and GCLC (**i**) were normalized to β-actin. The +PMA/-selenite/-LPS samples were set to 1. The data of (**a**–**c**,**e**,**g**–**i**) are given as means + SD (n = 3–4). Two-way ANOVA (**a**–**c**) or three-way ANOVA (**d**–**i**) with Bonferroni´s post-test. Significant outliers were determined by Grubbs´ test (α = 0.05). * *p* < 0.05, ** *p* < 0.01, *** *p* < 0.001 vs. cells without PMA (PMA effect); ^#^ *p* < 0.05, ^###^ *p* < 0.001 vs. cells without selenite (Se effect); ^§^ *p* < 0.05, ^§§^ *p* < 0.01, ^§§§^ *p* < 0.001 vs. cells without LPS (LPS effect).

Based on the observed changes in selenoprotein expression induced by PMA differentiation together with the well-known impact of many selenoproteins on the regulation of the cellular redox homeostasis [18,19], we wondered whether Se treatment affects the redox status in PMA-differentiated THP-1 macrophages. Surprisingly, total GSH levels decreased from PMA-induced differentiation independent of the Se status (Figure 3a). NRF2 and NF-κB are two transcription factors which are well-known to be regulated in a redox-dependent manner and to activate gene expression of antioxidant and inflammatory proteins, respectively [28]. The activity of NAD(P)H quinone oxidoreductase 1 (NQO1), a NRF2 target gene, was significantly upregulated in differentiated compared to undifferentiated cells (Figure 3b), which hints towards a putative activation of the transcription factor NRF2 in differentiated cells. To induce inflammatory conditions and activate the NF-κB signaling pathway, we included LPS stimulation for further experiments. NF-κB activation was studied by nuclear translocation of the subunit p65. In macrophages, NF-κB was activated by LPS stimulation in Se-treated but not in Se-deficient cells (Figure 3c). The mRNA expression of the NF-κB target genes COX2 and TNFα was increased by PMA-induced differentiation and further enhanced in LPS-stimulated THP-1 macrophages, with more pronounced effects under Se treatment (Figure 3d). Se effects were present in neither the release of TNFα nor COX2 protein levels (Figure 3e and Figure S3a). Moreover, proteins that are involved in the biosynthesis of prostaglandins (e.g., prostaglandin E synthase (PGES) and thromboxane (TX)A synthase 1 (TXAS1)), as well as leukotriens and resolvins (arachidonate 5-lipoxygenase (ALOX5) and arachidonate 15-lipoxygenase (ALOX15)), were upregulated by PMA but not affected by Se (Figure 3d). Interestingly, ALOX5 mRNA expression was significantly upregulated in undifferentiated THP-1 cells and downregulated in the differentiated cells by LPS. Otherwise, LPS did not significantly affect the protein expression of the two selenoproteins GPX4 and SELENOF (Figure S3c,d).

For mRNA levels, PMA treatment upregulated most NRF2 target genes (GCLC, GCLM, glutathione synthetase (GSS), HMOX1, NQO1, SOD1, TXNRD1, SRXN1) with the exception of SLC7A11, CAT, and GPX4 (Figure 3f). The strong PMA-dependent downregulation of SLC7A11, a subunit of the cystine/glutamate antiporter system X_c_^-^ that is essential for the intracellular supply with cysteine and subsequent GSH biosynthesis, might explain the decreased levels of GSH in the PMA-differentiated cells, although mRNA levels of the proteins of GSH biosynthesis (GCLC, GCLM, GSS) were upregulated. Notably, Se treatment makes THP-1 macrophages more responsive to LPS-induced NRF2 gene expression, with up to threefold higher mRNA levels of NRF2 target gene expression (e.g., HMOX1) under Se-treated conditions as compared to Se-deficient conditions. Protein expressions of NRF2 target proteins CAT, SOD1, and GCLC were not affected by LPS stimulation and Se treatment (Figure 3g–i). However, CAT expression was significantly downregulated and GCLC was upregulated by PMA, whereas no pronounced PMA effects were observed on SOD1 expression (Figure 3g–i).

### 2.4. Effects of LPS Treatment on LM profiles in THP-1 Monocytes and Macrophages

**Figure 4:**
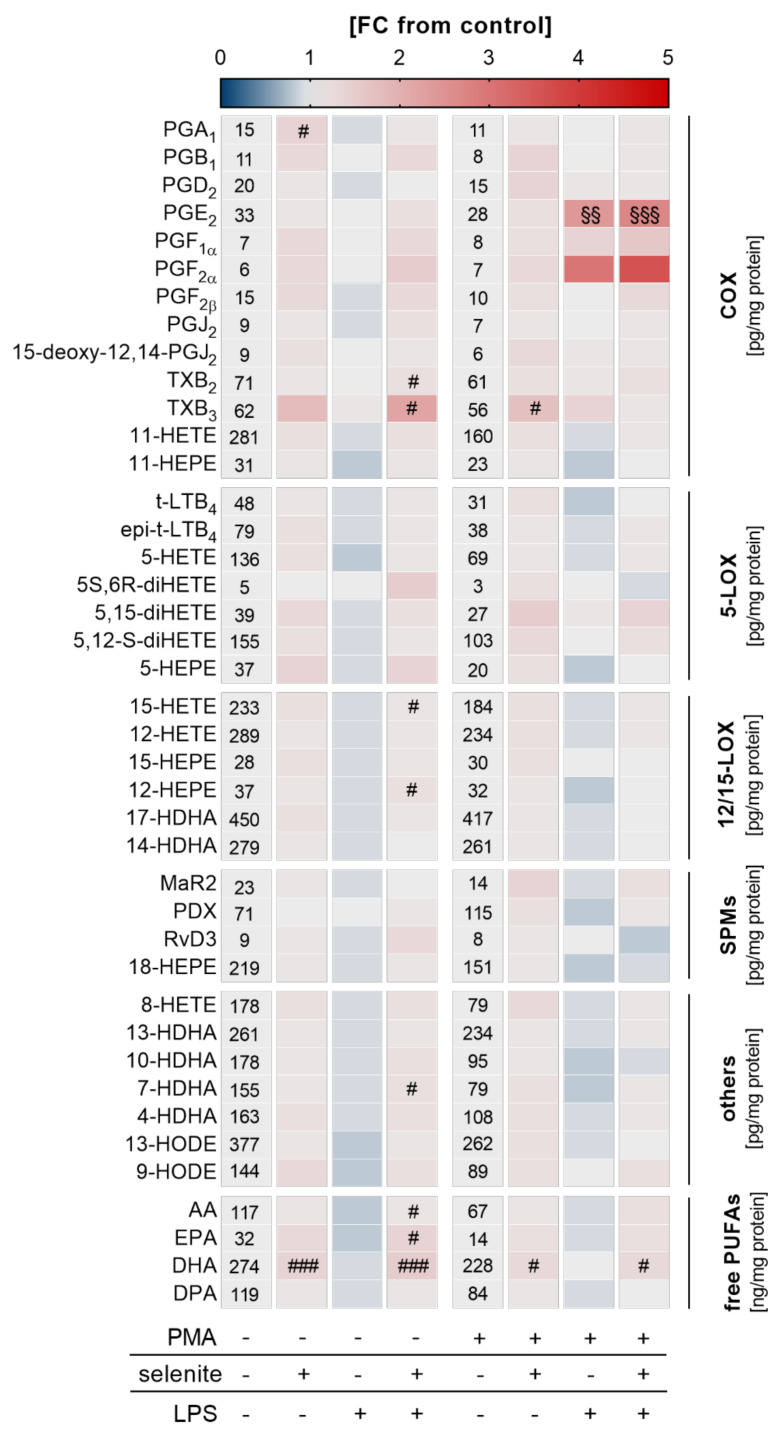
Effect of Se, PMA-induced differentiation, and LPS on lipid mediator (LM) profiles. THP-1 cells were pre-treated with or without 50 nM sodium selenite (selenite) for 72 h and differentiated into macrophages by treatment with 25 nM PMA in co-treatment with or without 50 nM sodium selenite (selenite) for 48 h and with or without 1 µg/mL lipopolysaccharide (LPS) for an additional 24 h. LM profiles of the supernatant were analyzed by UPLC-MS/MS. The heatmap organizes LM according to key biosynthetic enzymes (n = 4). The values in the columns give the concentrations of LMs in pg/mg protein (COX, 5-LOX, 12/15-LOX, SPMs, others) or ng/mg protein (free PUFAs). The color scale indicates the mean difference as fold change (FC) of control cells vs. LPS and/or selenite-treated cells (FC = 1). Two-way ANOVA with Bonferroni´s post-test. Significant outliers were determined by Grubbs´ test (α = 0.05). ^#^ *p* < 0.05, ^###^ *p* < 0.001 vs. cells without selenite (selenite effect); ^§§^ *p* < 0.01, ^§§§^ *p* < 0.001 vs. cells without LPS (LPS effect). AA, arachidonic acid; COX, cyclooxygenase; DHA, docosahexaenoic acid; DPA, docosapentaenoic acid; EPA, eicosapentaenoic acid; HDHA, hydroxy-docosahexaenoic acid; HEPE, hydroxypentaenoic acid; HETE, hydroxyeicosatetraenoic acid; HODE, hydroxyoctadecadienoic acid; LOX, lipoxygenase; LT, leukotriene; MaR, maresin; PD, protectin D; PG, prostaglandin; PUFA, polyunsaturated fatty acid; Rv, resolvin; SPM, specialized pro-resolving mediator; TX, thromboxane.

Given that Se-treated PMA-differentiated macrophages were more responsive to LPS-induced NF-κB activation, we speculated that an adequate Se status might also affect LM biosynthesis. A metabolipidomics approach was employed to investigate the impact of Se on LPS-induced changes on the LM profile. Similar to what we observed for mRNA expression of COX2 and TNFα (Figure 3d), nuclear translocation of the p65 subunit of NF-κB (Figure 3d), and the expression of NRF2 target genes (Figure 3f), Se supported the production of COX- and LOX-derived LMs as well as the release of the fatty acid substrates required for LM biosynthesis, reaching significance for the COX-derived LM TXB_2_ as well as docosahexaenoic acid (DHA) in differentiated macrophages. Interestingly, in undifferentiated Se-deficient cells, almost all lipid mediators were downregulated by LPS by trend but upregulated by Se with significant effects on COX-derived TXB_2_ and TXB_3_, and the LOX-derived products 15-hydroxyeicosatetraenoic acid (15-HETE) and 12-hydroxypentaenoic acid (12-HEPE), as well as arachidonic acid (AA), eicosapentaenoic acid (EPA), and docosahexaenoic acid (DHA) (Figure 4). Together, our data clearly indicate that Se facilitates LM biosynthesis by modulating the enzymes for LM biosynthesis.

## 3. Discussion

Since Se was recognized as an essential trace element, the role played by its adequate supply has been intensively studied. Various studies have shown that adequate levels of Se are important for the immune function and are able to prevent excessive inflammation [3]. Hence, Se was found to limit the expression of pro-inflammatory cytokines under inflammatory conditions in vitro at relatively high concentrations of 2 µM [29,30,31]. In vivo effects of Se are more distinct [32,33]. While long-term treatment of mice with 0.6 mg/kg Se (which was given as sodium selenite and corresponded to approximately four times the adequate intake) had no effects on dextran sulfate sodium (DSS)-induced colitis, short-term treatment with 0.6 mg/kg Se actually enhanced inflammation scores, as well as TNFα and COX2 mRNA expression. Notably, 0.6 mg/kg Se given in the form of selenomethionine had no effect [32]. Otherwise, rats that received 0.2–20 µg/kg organic selenocompounds during pregnancy developed less severe *Staphylococcus aureus*-induced mastitis with lower NF-κB activation and TNFα mRNA expression [33]. Within the innate immune system, macrophages inherit key functions in neutralizing pathogens and regulating inflammatory processes and individual selenoproteins have been shown to affect the macrophage maturation process (GPX1 and SELENOP) and limit oxidative burst (SELENOK) [34,35,36,37,38]. However, little is known about how Se deficiency impacts redox-dependent proliferation, differentiation and signaling cascades in monocytes and differentiated macrophages, nor about how selenoprotein expression affects these processes.

Human THP-1 monocytes were used as a model system under Se-deficient as well as Se-adequate conditions and were differentiated into macrophages by PMA. Interestingly, the hierarchy of selenoprotein expression seems to be different in monocytes/macrophages as compared to other cell types, such as epithelial cells. While the Se-sensitive selenoprotein SELENOH was significantly upregulated in a time- and concentration-dependent manner, with fourfold higher expression levels at 500 nM Se, the expression of another Se-sensitive selenoprotein, GPX1, was only slightly affected by Se, being upregulated by 1.5-fold (Figure S1a,b). Notably, the protein expression of GPX1 and of the protein family member GPX4, which ranks higher in hierarchy, were comparably upregulated by 1.5–1.8-fold by Se, which indicates that GPX1 is more indispensable in THP-1 monocytes compared to other cell types (Figure 2a and Figure S2a). This might be due to the absence of another GPX isoenzyme in monocytes, GPX2 [35], which has been previously shown to have overlapping functions with GPX1 in limiting the redox-dependent activation of the NF-κB pathway and LM biosynthesis in human epithelial-derived cancer cells [29]. It is therefore tempting to speculate that the absence of the epithelial GPX2 makes GPX1 more indispensable in THP-1 monocytes, thereby advancing GPX1 in hierarchy.

Interestingly, the PMA-induced differentiation process was also not significantly affected by Se status, as suggested from cell adherence, granularity, autofluorescence, and surface marker expression (Figure 1b–e). However, the differentiation process had a strong impact on the protein expression levels of various selenoproteins (Figure 2 and Figure S2). Expression of the two GPX isoenzymes GPX1 and GPX4 was differently regulated by PMA. Whereas GPX1 protein expression was not affected by PMA treatment (Figure S2a), GPX4 protein expression was downregulated under both Se-deficient as well as Se-adequate conditions by PMA-induced differentiation (Figure 2a). Notably, mRNA expression levels of selenoproteins did not correlate with protein expression levels and were upregulated by PMA treatment for all selenoproteins except for SELENOO (Figure 2e). We could exclude (i) Se levels, (ii) impaired selenocysteine biosynthesis, and (iii) selenoprotein translation as limiting variables for the PMA-induced reduction of several selenoproteins since the cellular Se content as well as mRNA expression of proteins responsible for selenocysteine biosynthesis and selenoprotein translation were not affected or even upregulated by PMA (Figure 2e,f). We therefore speculate that the upregulation of selenoprotein mRNAs is due to the special M0 state of macrophages, which has to be considered as a transitional rather than a terminal stage. Depending on the milieu in the tissue, M0 macrophages polarize into various subpopulations with either pro- or anti-inflammatory properties. Hence, mRNA levels of the selenoproteins might be kept at relatively high levels to rapidly adapt the protein expression of selenoproteins to the requirement of the respective macrophage subtype after maturation.

GPX4 has been shown to inhibit NF-κB activation [39], and the downregulation of GPX4 in differentiated THP-1 macrophages might be essential to enable inflammatory responses in these cells. Accordingly, NF-κB target genes TNFα and COX2 were upregulated by PMA (Figure 3d). Moreover, TNFα and COX2 mRNA expression as well as the release of COX-derived PGE_2_ and PGF_2α_ were increased by LPS stimulation in macrophages, with more distinct effects under Se treatment (Figure 3 and Figure 4). However, the Se effect on TNFα and COX2 expression was not present for protein levels (Figure 3e and Figure S3a). Possibly, Se effects on protein expression are only visible within another time frame which was not captured by our study (24 h). Essentially, previous studies indicate that the Se effects on biosynthesis of pro-inflammatory cytokines differ at different time points [30,40]. Importantly, a rapid and adequate secretion of inflammatory cytokines upon bacterial or viral infection is essential in pathogen removal and to prevent sustained inflammation. Optimal Se supply might be important to maintain homeostasis between the formation of inflammation-dependent production of reactive oxygen species and the antioxidant NRF2 system, thereby enabling cells’ fast upregulation of inflammatory response upon stimulation, and to prevent chronic inflammation [41]. Inversely, physiological changes in Se levels as studied herein only moderately impacted on NF-κB signaling and LM biosynthesis, which indicates that THP-1 cells are able to compensate for the reduced expression of selenoproteins. Otherwise, expression of selenoproteins that are important for the regulation of inflammatory signaling might be less dependent on Se in macrophages, as demonstrated for GPX1 and TXNRD1 (Figure S2a,c).

Interestingly, the release of other COX-derived products was enhanced neither by PMA nor by LPS (Figure 4), which indicates that LM biosynthesis is efficiently channeled into PGE_2_ and PGF_2α_ production. Notably, previous studies reported that Se treatment decreased the expression of pro-inflammatory cytokines such as TNFα and the release of LMs including PGE_2_ in macrophages [29,30,31,33,40,42,43]. Differences in the study outcomes might have been related to different experimental settings. While our study used human THP-1 macrophages treated with physiological concentrations of Se (50 nM), previous studies employed murine RAW264.7 and bone marrow-derived macrophages (BMDM) treated with 2 µM Se (given as sodium selenite). Hence, even striking differences in the Se-dependent regulation of COX2 expression are observed in RAW264.7 and BMDM cells under similar experimental conditions (2 µM Se), with strong effects of Se (2 µM) on COX2 expression in RAW264.7 and noticeably less pronounced effects in the BMDM cells [30]. Moreover, another study showed that TNFα mRNA expression in *Staphylococcus aureus-*stimulated RAW264.7 macrophages was significantly reduced by 2 µM Se (given as sodium selenite) and unaffected by 1–1.5 µM [40], which underlines the concentration dependency of the Se effects. Notably, while an adequate supply with Se is supposed to have antioxidant properties via the upregulation of selenoproteins, Se given at concentrations that exceed the dosage required to maximize selenoprotein expression most likely does not exert its effects via selenoproteins and can even have pro-oxidant effects, which underscores the necessity of a well-balanced Se status [44,45,46]. Another critical aspect of an appropriate immune reaction might be that related to an accurate time-dependent regulation of the inflammatory response. While an upregulation of COX2 expression after infection is required to remove pathogens, an excessively prolonged activation might result in chronic inflammation. Overall, it appears that moderate changes in the Se supply do not overtly change the expression of NF-κB-responsive proteins and thus the immune response of THP-1 monocytes and M0 macrophages.

Analog with NF-κB, mRNA expression levels of NRF2 target genes were increased by LPS stimulation and were dependent on adequate Se levels in macrophages (Figure 3f). The increase of NRF2 target gene transcription by LPS is in line with a previous report which also observed an upregulation of NRF2 upon activation of the toll-like receptor 1/2 with Pam3CSK4 in THP-1 cells [47]. Given that the NF-κB and NRF2 signaling pathways are interconnected via a complex signaling network, the coordinated increase in NF-κB and NRF2 target gene expression upon LPS stimulation by Se hints towards an important function in maintaining a balance of NF-κB-dependent reactive oxygen species/reactive nitrogen species formation and NRF2-dependent hydrogen peroxide detoxification in THP-1-derived macrophages. Otherwise, Se downregulated most of the NRF2 target genes under unstimulated conditions in macrophages, reaching significance for GCLC (Figure 3f). This is in line with a previous in vivo study which found that mRNA expression of the NRF2 target genes GCLC, NQO1, SOD1, and SRXN1 was significantly downregulated in mice that received a Se-adequate diet (0.15 mg/kg) compared to mice with a Se-poor diet (0.086 mg/kg) [48]. Notably, while most of the NRF2 target genes were upregulated by PMA-induced differentiation, the two target genes CAT and SLC7A11 were downregulated (Figure 3f). NRF2-dependent target gene expression is regulated by complex mechanisms, which include: (i) different cysteine sensors within the NRF2 regulatory protein KEAP1 (Cys151, Cys257, Cys273, Cys288, and Cys297) that can be targeted by distinct endogenous and exogenous ligands, (ii) phosphorylation of NRF2, and (iii) transactivation via dimerization with different transcription factors (e.g., activating transcription factor 4 (ATF4), ATF3 or CREB binding protein (CBP)) and nuclear receptors (e.g., peroxisome proliferator-activated receptor gamma (PPARγ), estrogen receptor α, and retinoic X receptor α (RXRα)) [15]. How exactly PMA stimulates NRF2 activation is not known. However, a similar controversial regulation of NRF2 target gene subsets has also been observed with other small molecule inducers of NRF2, such as Bay 11-7085 [49] and sulforaphane [50], which stimulate ferroptosis and induce a strong upregulation of HMOX1 with concomitant downregulation of other NRF2 target genes like GPX4 and SLC7A11, respectively. Depending on the point of attack, small molecule inducers of NRF2 might therefore reveal a specific profile of NRF2 target gene regulation.

In conclusion, we anticipate that adequate Se levels as well as differentiation impact on selenoprotein expression, whereas the Se status only moderately affected NF-κB signaling and biosynthesis of inflammatory LMs. Of particular interest are the discrepancies with previous findings that indicated an inhibitory effect of Se on NF-κB-dependent inflammatory signaling; our results indicate that Se facilitates LPS-induced NF-κB activation. We speculate that differences arise from (i) different Se concentrations and (ii) timing and maybe even the species (e.g., human vs. mouse). Notably, PMA, which was used for differentiation, had significant effects on NF-κB as well as NRF2 that might have impacted the overall effects of Se in our THP-1 model system. However, the well-known effects of physiological Se levels for a fine-tuned adaptive immune response upon bacterial and viral infections [51,52], as well as during inflammation resolution [53,54,55], might be mediated by other mechanisms as well. Further concentration- and time-dependent studies on inflammatory responses upon inflammatory stimulation are required, as are investigations in primary cells which more closely reflect in vivo conditions and research on the function of specific selenoproteins in order to address these issues.

## 4. Materials and Methods

### 4.1. Materials

Materials are listed in Table S1.

### 4.2. Cell Culture

Human THP-1 acute monocytic leukemia cells (monocytes, ATCC^®^ TIB-202™) were grown in RPMI completed with 10% (*v/v*) FCS, 1% (*v/v*) penicillin-streptomycin and 1% GlutaMAX at 37 °C in 5% CO_2_ and sub-cultured every three to five days. Cell treatment: THP-1 monocytes were incubated with different concentrations of Se (0–500 nM) for 72 h or for varying incubation times (0–96 h) with 50 nM Se. THP-1 monocytes were differentiated with different PMA (0–400 nM) concentrations as indicated or with 25 nM PMA for 48 h or as indicated. THP-1 monocytes were preincubated for 72 h with or without 50 nM Se, seeded at densities of 0.5 × 10^5^ cells/mL THP-1 monocytes (DMSO control) and 2.5 × 10^5^/mL for PMA-induced differentiation with or without Se (50 nM) treatment, and harvested after 48 h. LPS (1 µg/mL) stimulation was performed with undifferentiated and differentiated THP-1 cells with or without Se treatment for 1 h (nuclear extracts for Western blot analysis), 6 h (quantitative real-time PCR analysis) or 24 h (Western blot and LM analysis). The protein content of the cell lysates was measured using Bradford analysis (Bio-Rad Laboratories).

### 4.3. Determination of Adherence, Morphology and Cell Number

Adherence of PMA-treated THP-1 cells was determined at the indicated time points. Cells were harvested by trypsinization and resuspended in RPMI. The cell number was determined using 0.4% trypan blue staining with a Vi-Cell XR Cell Viability Analyzer (Beckman Coulter, Brea, CA, USA). To determine the relative adherence, adherent cells were collected and percentage adherence was calculated as the number of adherent cells divided by the starting cell number and multiplied by 100.

Images of cells were collected with an Eclipse Ti-S microscope (Nikon GmbH, Tokyo, Japan) equipped with a Nikon Digital Sight DS-Fi1c color camera (Nikon GmbH) and a 20× objective (NA = 0.45, S Plan Fluor, Nikon GmbH) or a 10× objective (NA = 0.3, Plan Fluor, Nikon GmbH).

### 4.4. Flow Cytometry

For flow cytometry experiments, cells were treated as described above and harvested. Granularity was measured using forward and side scatter light and autofluorescence analyzed in unstained cells in the FL-1 channel (blue laser, excitation 488, emission 525/40). For detection of the cell surface marker CD68, cells were resuspended in ice cold primary antibody solution (containing phosphate-buffered saline (PBS, 140 mM NaCl, 10 mM Na_2_HPO_4_, and 2.99 mM KH_2_PO_4_, pH 7.4) with 10% FCS and 1% NaN_3_), fixed with PBS and 0.01% formaldehyde (diluted in PBS) for 15 min, and washed with primary antibody solution. Cells were resuspended in primary antibody solution with 0.5% (*v/v*) Tween^®^ 20 and incubated with primary antibody mouse anti-CD68 (1:200) for 30 min. Samples were centrifuged for 3 min at 500*× g* and washed twice in primary antibody solution and once in secondary antibody solution (containing PBS with 3% BSA and 1% NaN_3_) with the same centrifugation steps in between. Samples were incubated with the secondary antibody (anti-mouse IgG Alexa Fluor^®^ 594 Conjugate (1:1000)) for 30 min protected from light. Analysis was performed on a CytoFLEX flow cytometer (Beckman Coulter, excitation: 488 nm; emission: 525/40 nm) and data were analyzed using CytExpert software version 2.2 and version 2.3 (Beckman Coulter).

### 4.5. Nuclear Isolation

The nuclear lysates of 3.0 × 10^5^/mL PMA-differentiated macrophages were harvested after 1 h of LPS stimulation. Lysis buffer I (10 mM HEPES, 1.5 mM MgCl_2_, 10 mM KCl, 0.5 mM DTT, 0.5 mM PMSF, and 0.1% NP-40 Alternative, pH 7.9) was used. After incubation for 7 min at 4 °C under shaking, cells were centrifuged for 1 min at 4 °C and 6800× *g*. Thereafter, NaCl (5 M) was added to the lysis buffer II (40 mM HEPES, 400 mM KCl, 10% glycerol, 1 mM DTT, and 0.1 mM PMSF, pH 7.9) and the cell pellet was lysed by ultrasonic treatment (80% amplitude, 0.5 s cycle), and centrifuged for 30 min at 4 °C and 20,000*× g* to obtain nuclear lysates. The protein content was measured and the nuclear lysate was used for Western blot analysis.

### 4.6. Western Blot

Harvested THP-1 monocytes and macrophages were lysed with RIPA buffer (50 mM Tris, 150 mM NaCl, 2 mM EDTA, 0.5% sodium deoxycholate, 0.1% SDS, and 1% NP-40 Alternative, pH 7.7 with 0.1% (*v/v*) protease inhibitor) for 15 min at 4 °C and 1200 rpm using the ThermoMixer^®^ (Eppendorf AG, Hamburg, Germany). The samples were centrifuged for 15 min at 4 °C and 14,000× *g*, and protein concentration was determined in the supernatant. Samples were mixed with loading buffer (1×; 41.7 mM Tris pH 6.8, 10% glycerin, 2% SDS, 0.125% bromophenol blue, and 2.5% β-mercaptoethanol) and heated for 5 min at 95 °C. Proteins were separated on SDS polyacrylamide gels (10–15%) and immunoblotted on Amersham™ Protran^®^ nitrocellulose membrane. Membranes were incubated for 2 min in Ponceau S solution (0.2% (*w/v*) with 3% (*w/v*) trichloroacetic acid) and bands were recorded by a ChemiDoc^TM^ MP Imaging System (Bio-Rad Laboratories). Subsequently, membranes were blocked in 5% (*w/v*) non-fat dry milk in Tris-buffered saline (5 mM Tris, 15 mM NaCl) with 0.1% (*v/v*) Tween^®^ 20 (T-TBS) for 1 h at room temperature and incubated with primary antibodies overnight at 4 °C in T-TBS: Mouse anti-γ-GCSc (H-5) (1:10,000), mouse anti-mPGES1 (1:300); rabbit anti-beta actin (1:10,000); rabbit anti-oxidative stress defense (catalase, SOD1) (1:500); rabbit anti-COX2 (D5H5) XP^®^ (1:1000); rabbit anti-GPX1 (1:5000); rabbit anti-GPX4 (1:5000); rabbit anti-NF-κB p65 (D14E12) XP^®^ (1:4000); rabbit anti-p21WAF1/Cip1 (12D1) (1:1000); rabbit anti-SEP15 (1:3000); rabbit anti-SELH (1:1000); rabbit anti-SELO (1:2500); rabbit anti-VIMP (D1D1M)) (1:1000); rabbit anti-TXNRD1 (1:10,000); and rabbit anti-TXNRD2 (1:1000). HRP-coupled goat anti-rabbit IgG (1:50,000) or HRP-coupled horse anti-mouse IgG (1:3000) were used as secondary antibody. Protein bands were detected using SuperSignal^TM^ West Dura and band intensities were densitometrically quantified using a ChemiDoc^TM^ MP Imaging System (Bio-Rad Laboratories) and data analyzed using Image Lab software version 5.0 (Bio-Rad Laboratories). Protein expression was normalized to ß-actin or Ponceau S staining as indicated.

### 4.7. Quantitative Real-Time PCR

Total mRNA of THP-1 cells was isolated using the Dynabeads™ mRNA DIRECT™ Purification Kit according to the manufacturer’s protocol. The SensiFAST™ cDNA Synthesis Kit was used for cDNA transcription. For real-time PCR, cDNA were combined with Master mix (PerfeCTa SYBR Green Supermix, forward and reverse primer (each 250 nM final concentration)) in a 96-well plate as previously described [56]. Primer sequences are given in Table 1. PCR was performed on a real-time PCR system (MX3005P, Agilent, Santa Clara, CA, USA) with heat cycle to 95 °C for 3 min, 40 cycles of denaturation at 95 °C for 15 sec, annealing at 60 °C for 20 s, and elongation at 72 °C for 30 sec. Standard curves from diluted PCR products were used for quantification and all samples and standards were measured in triplicate. For normalization, sample values were normalized to a composite factor based on the reference genes RPL13a and RPS9. MIQE guidelines served as reference for the quantification procedure.

### 4.8. ELISA

Incubation medium from seeded cells was collected and the content of TNFα was determined according to the manufacturer’s protocol using a microplate reader (Synergy H1, BioTek, Bad Friedrichshall, Germany). A Human TNFα Pre-Coated ELISA Kit was used. The incubation medium of THP-1 monocytes (DMSO control) was diluted 1:2 and for PMA-induced differentiated macrophages 1:50. A standard curve was used to generate a four-parameter logistic curve-fit and to calculate the content of TNFα which was normalized to the protein content of the cells.

### 4.9. Trace Element Analysis by Total Reflection X-ray Fluorescence (TXRF) Spectroscopy

TXRF was used to determine the intracellular levels of Se. Harvested THP-1 monocytes and macrophages were collected and lysed as described in Section 4.6. Cellular debris was removed by centrifugation for 10 min at 4 °C and 15,000× *g*. Trace elements analysis was performed using the supernatant (S2 Picofox™, Bruker Nano GmbH, Berlin, Germany), and 1 mg/L yttrium was used as internal standard. Ten microliters of each sample was placed on a siliconized quartz glass carrier and dried for 30 min at 40 °C. Samples were measured randomized in triplicate for 1000 s respectively. The Se content was normalized to the protein content of the samples.

### 4.10. GSH Assay

For the measurement of the total GSH content, cells were harvested, resuspended in 150 µL ice-cold 10 mM HCl, and lysed by ultrasonic treatment (80% amplitude, 0.5 s). Cellular debris was removed by centrifugation for 30 s at 8000*× g* at room temperature. Then, 30 µL (*w/v*) 5% SSA was added to the supernatant, incubated for 10 min, and centrifuged for 15 min at 4 °C and 8000*× g* at 4 °C, and supernatant was used for GSH measurement. After addition of 10 mM DTNB, the formed 2-nitro-5-thiobenzoic acid (TNB) was measured on a microplate reader (Synergy H1) at 412 nm for 5 min with path-length correction, as previously described [57]. BSO (0.25 mM) was used as control for GSH depletion and added 24 h before the cell harvest. A standard curve was used to calculate the total GSH content, which was normalized to the protein content of the samples.

### 4.11. Enzyme Activities

Cells were homogenized with a TissueLyser II (Qiagen, Hilden, Germany) in Tris buffer (100 mM Tris, 300 mM KCl, pH 7.6 with 0.1% (*v/v*) Triton X-100, and 0.1% (*v/v*) protease inhibitor) twice for 60 s at 30 Hz and centrifuged for 10 min at 4 °C and 14,000× *g*. The enzymatic activity was normalized to the protein content of the samples.

NQO1 activity was measured via menadione-dependent reduction of MTT as previously described [58]. Briefly, cell lysates were mixed with reaction buffer. Incubation mixtures contained 25 mM Tris, pH 7.4, 0.665 mg/l BSA, 0.01% Tween^®^ 20, 5 µM FAD, 1 mM D-glucose-6-phosphate, 35 µM NADP, 0.72 mM MTT, 0.3 U/mL glucose-6-phosphate dehydrogenase, and 50 µM menadione. Absorbance was measured at 590 nm for 5.5 min using a microplate reader (Synergy H1) with path-length correction. Differences between the MTT reduction rates with and without dicumarol (final concentration 60 µM in phosphate buffer (1 mM KH_2_PO_4_, pH 7.4 and 0.1% DMSO) were used for calculation of NQO1 activity (ε_590 nm_ = 11.961 mM^−1^ × cm^−1^). Data are given as mUnit(mU)/mg protein.

GPX activity was measured by a GR-coupled test with NADPH consumption using H_2_O_2_ as substrate. Briefly, cell lysate was mixed with reaction buffer. Incubation mixtures contained Tris buffer (96 mM Tris, 4.8 mM EDTA, 960 µM NaN_3_, pH 7.6) with 0.11% Triton X-100, 224 µM NADPH, 3.37 mM GSH, and 78 mU/mL GR and were incubated for 10 min at 37 °C. Ten microliters H_2_O_2_ (0.00375%) was added and the reaction was monitored for 2 min at 340 nm and 37 °C using a microplate reader (Synergy H1) with path-length correction. Data are given as mU/mg protein (ε_340 nm_ = 6.22 mM^−1^ × cm^−1^).

TXNRD activity was assessed using DTNB. Cell lysates were mixed with reaction buffer. Incubation mixtures contained 185 µL phosphate buffer (92.5 mM KH_2_PO_4_, 1.85 mM EDTA, pH 7.4) and 15 µL DTNB (3.75 mM in DMSO)). Twenty-five microliters NADPH (final concentration of 200 µM in phosphate buffer) was used to start the reaction, and TNB formation was monitored at 412 nm for 5 min using a microplate reader (Synergy H1) with path-length correction. TXNRD-independent formation of TNB was determined in the absence of NADPH and was subtracted. Data are given as mU/mg protein (ε_412 nm_ = 13.6 mM^−1^ × cm^−1^).

### 4.12. Solid Phase Extraction of LMs

LMs were extracted from cell culture medium as previously described [29]. Briefly, medium was mixed with a twofold amount of ice-cold methanol containing deuterium-labeled internal standards (200 nM d8-5S-HETE, d4-LTB4, d5-LXA_4_, d5-RvD_2_, d4-PGE_2_, and 10 μM d8-AA). Proteins were precipitated at −20 °C overnight and clear supernatant was collected after centrifugation (10 min, 1200× *g*, 4 °C). LMs were extracted from supernatant as previously described [59]. Briefly, the supernatant was acidified with water, pH 3.5, and LMs were purified by solid phase extraction (Sep-Pak^®^ Vac 6 cc 500 mg/6 mL C18; Waters, Milford, CT, USA) using MilliQ water and n-hexane for washing steps. LMs were eluted with methyl formate, eluates were evaporated to dryness using nitrogen (TurboVap LV, Biotage, Uppsala, Sweden), and LMs were resolved in 100 µL methanol/water (50/50) for UPLC-MS/MS analysis [29].

### 4.13. LM Analysis by UPLC-MS/MS

Separation of LMs was performed at 50 °C on an Acquity UPLC^®^ BEH C18 column (1.7 μm, 2.1 × 100 mm; Waters, Eschborn, Germany) with an Acquity™ UPLC system (Waters) as described previously [29]. In brief, methanol-water-acidic acid was used as mobile phase starting at a ratio of 42:58:0.01, which was gradually changed to 86:14:0.01 over 12.5 min and then to 98:2:0.01 over 3 min using a flow rate of 0.3 mL/min. LMs were analyzed on a QTRAP 5500 ESI tandem mass spectrometer (AB Sciex, Darmstadt, Germany) which was coupled to the LC system. After electrospray ionization operated in negative mode, multiple reaction monitoring was performed in the negative ionization mode with parameters adjusted as previously described [59]. External standards were used to confirm the retention time, and calibration curves for each analyte were prepared. For quantification of LMs, analyte levels were normalized by calculating the ratio of internal standard and directly measured deuterium-labeled standards to account for loss of analytes during purification and measurement, as reported previously [60], and they were normalized to the protein content of the cells.

### 4.14. Statistics

Data are given as means + SD of independent experiments. The statistical analysis was performed with GraphPad Prism 8.4.3 Software (San Diego, CA, USA) using one-way, two-way or three-way analysis of variance (ANOVA) with Bonferroni´s post-test. Outliers were determined by Grubbs’ test, with significance level α = 0.05. *p*-values < 0.05 (^*^), (^#^), (^§^), (^&^), (^$^), (^+^); *p* < 0.01 (^**^), (^##^), (^§§^), (^&&^), (^$$^), and for *p* < 0.001 (^***^), (^###^), (^§§§^), (^&&&^), (^$$$^), (^+++^) were considered as statistically significant. Figures were created with GraphPad Prism 8.4.3 Software.

## Figures and Tables

**Table 1:** Human-specific primers for quantitative real-time PCR.

Gene	RefSeq-ID	Sequence (5′→3′)
ALOX15 (arachidonate 15-lipoxygenase)	NM_001140.3	TGGAGCCTTCCTAACCTACAG TCCACATACCGATAGATGATTTCC
ALOX5 (arachidonate 5-lipoxygenase)	NM_000698.3	GCTGCAACCCTGTGTTGATCC AAATGTTCCCTTGCTGGACCTC
CAT (catalase)	NM_001752.4	CCTATCCTGACACTCACCGCCA GAGCACCACCCTGATTGTCCTG
COX2 (cyclooxygenase 2; prostaglandin-endoperoxide synthase 2 (PTGS2))	NM_000963.2	CCCAGCACTTCACGCATCAG CTGTCTAGCCAGAGTTTCACCGT
EEFSEC (eukaryotic elongation factor, selenocysteine-tRNA-specific)	NM_021937.3	CCCTAGAGAACACCAAGTTCCGAG TCAATGAGCTCTGGAATGCCCT
EIF4A3 (eukaryotic translation initiation factor 4A3)	NM_014740.3	AAAGAAAGGTGGACTGGCTGACGG ACTCCTTCATGATGGACTCCCGCT
GCLC (glutamate-cysteine ligase catalytic subunit)	NM_001498.3	TGCTGTCTCCAGGTGACATTCCA GGAGATGCAGCACTCAAAGCCA
GCLM (glutamate-cysteine ligase modifier subunit)	NM_002061.3	GTTGACATGGCCTGTTCAGTCCT CCCAGTAAGGCTGTAAATGCTCCA
GPX1 (glutathione peroxidase 1)	NM_000581.2	TACTTATCGAGAATGTGGCGTCCC TTGGCGTTCTCCTGATGCCC
GPX3 (glutathione peroxidase 3)	NM_002084.5	GTCGAAGATGGACTGCCATGGT AGCTGGCCACGTTGACAAAGAG
GPX4 (glutathione peroxidase 4)	NM_002085.3	AGGCAAGACCGAAGTAAACTACAC TCTCTTCGTTACTCCCTGGCT
GSS (glutathione synthesis)	NM_000178.2	CCAAGTGCCCAGACATTGCCA CCTTCTTCACCCACATCCAGTGAG
HMOX1 (heme oxygenase 1)	NM_002133.2	CAACAAAGTGCAAGATTCTGCCC CTACAGCAACTGTCGCCACC
NOX2 (NADPH oxidase 2)	NM_000397.3	TCACCAAGGTGGTCACTCACCC TGCCACTCCAGCTTGGACAC
NQO1 (NAD(P)H quinone oxidoreductase 1)	NM_001025434.1	CATCACAGGTAAACTGAAGGACCC CTCTGGAATATCACAAGGTCTGCG
PGES (prostaglandin E synthase)	NM_004878.3	ACGCTGCTGGTCATCAAGATG TGGCAAAGGCCTTCTTCCGC
PSTK (phosphoseryl-tRNA kinase)	NM_153336	TTTGAGGCCCAGTCTTGCTACC GCCCAACGAATATTTCCGAGCC
RPL13a (ribosomal protein L13a)	NM_012423.4	GAGGTTGGCTGGAAGTACCAGG TGTTTCCGTAGCCTCATGAGCTG
RPS9 (ribosomal protein S9)	NM_001013.4	CCATATCAGGGTCCGCAAGCA GGCCCTTCTTGGCATTCTTCCT
SECISBP2 (SECIS-binding protein 2)	NM_024077.4	TGAAGAGCCACCAGGCACAG GCATCTGGCTGCAGTAATCCCT
SELENBP1 (selenium-binding protein 1)	NM_009150.3	TTCCCTTGGAGATCCGCTTCCT ACTGACCATGTACCTCCCTCGT
SELENOF (selenoprotein F)	NM_203341.1	TGATCTTCTCGGACAGTTCAACCT CACGGACATACTTGGACTTGAGGG
SELENOH (C11orf31, chromosome 11 open reading frame 31; selenoprotein H)	NM_170746.2	GCTTCCAGTAAAGGTGAACCCGA TCAGGGAATTTGAGTTTGCGTGG
SELENOI (selenoprotein I)	NM_033505.4	ATGCCTCAGCACCAGGTCAC GTTCTGCGAGCTTGCTTTCCGT
SELENOK (selenoprotein K)	NM_021237.3	GATGATGGAAGAGGGCCACCAG CGCATGTCCGGTTGTCTGCT
SELENOM (selenoprotein M)	NM_080430.2	TGAAGGCTTTCGTCACGCAG AGTGGGATGCGCTCTAGTTCCT
SELENON (selenoprotein N)	NM_206926.2	TGTGATGTTCCGGATCCATGCC TGTGGTTGGGCACGAAGAGC
SELENOO (selenoprotein O)	NM_031454.1	TGACGCCGAGTTCCAAAGGCA TTGTGAAGTCGGCACCGGTCAG
SELENOP (selenoprotein P)	NM_005410	GAAACTCCATCGCCTCATTACCAT CTGCCTATGCTGACCCTTGTG
SELENOS (selenoprotein S, VIMP)	NM_203472.1	GCTGCATCCTTCTCTACGTGGTC CAACAACATCAGGTTCCACAGCA
SELENOT (selenoprotein T)	NM_016275.3	CGATCATAGCACCACCTATCAGCA GAGCCTGCCAAGAAAGCATCTG
SEPHS2 (selenophosphate synthetase 2)	NM_012248.4	GACGGTTTGGGCTTCTTCAAGG TCCACAATGCCAACGATCCAC
SEPSECS (sep (o-phosphoserine) tRNA:sec (selenocysteine) tRNA synthase)	NM_016955.3	CTAGTGCTCCCGCTTATTCGCC CTGGACACTTGCCCTTCTCCAG
SLC7A11 (solute carrier family 7 member 11)	NM_014331.3	TGCTCTTCTCTGGAGACCTCGAC ACAGTGGCACCTTGAAAGGACG
SOD1 (superoxide dismutase 1)	NM_000454.4	TACAGCAGGCTGTACCAGTGCA TCGGCCACACCATCTTTGTCAG
SRXN1 (sulfiredoxin 1)	NM_080725.1	CTCAGTGCTCGTTACTTCATGGTC GTTTGGCCCTTCCTCTTCCTCC
TNFA (tumor necrosis factor α)	NM_000594.2	AGCCCATGTTGTAGCAAACCCT GGAGTAGATGAGGTACAGGCCC
TXAS1 (thromboxane A synthase 1)	NM_001061.4	CCTGAAAGGTTCACGGCTGAG CAACTTGACCTCAAGCAGCCCT
TXNRD1 (thioredoxin reductase 1)	NM_182742.1	GTGTTGTGGGCTTTCACGTACTG TGTTGTGAATACCTCTGCACAGAC
TXNRD2 (thioredoxin reductase 2)	NM_006440.5	GTTCCCACGACCGTCTTCAC GTGATAGACCTCAACATGCTCCTG

## Data Availability

The data presented in this study are available in Supplementary Materials.

## Supplementary Materials

The following are available online at https://www.mdpi.com/article/10.3390/ijms222011060/s1.
