# Supplementary material for: The Trace Element Selenium Is Important for Redox Signaling in Phorbol Ester-Differentiated THP-1 Macrophages
Source: Int J Mol Sci. 2021 Oct 14;22(20):11060. doi: 10.3390/ijms222011060 (PMC8539332; doi:10.3390/ijms222011060)
Supplement: Supplementary file 1 [file ijms-22-11060-s001.zip › ijms-1400884-supplementary.pdf]

Supplementary Materials

Table S1. Used material

Material	Company
Primary antibody mouse anti-CD68 (ab201340)<br>Primary antibody rabbit anti-beta actin (ab8227)<br>Primary antibody rabbit anti-GPX4 (ab125066)<br>Primary antibody rabbit anti-oxidative stress defense (catalase, SOD1, TRX, smooth muscle actin) (ab179843)<br>Primary antibody rabbit anti-SELH (selenoprotein H) (ab151023)<br>Primary antibody rabbit anti-SELO (selenoprotein O) (ab172957)<br>Primary antibody rabbit anti-SEP15 (selenoprotein F) (ab124840)<br>Primary antibody rabbit anti-TXNRD1 (ab124954)<br>Primary antibody rabbit anti-TXNRD2 (ab180493)	Abcam<br>(Cambridge, UK)
5,5-dithio-bis-(2-nitrobenzoic acid) (DTNB)<br>Bovine serum albumin (BSA)<br>Potassium chloride (KCl)<br>Sodium bicarbonate ($NaHCO_3$)<br>Sodium dodecyl sulfate (SDS) ultrapure	Applichem<br>(Darmstadt, Germany)
Human TNFα Pre-Coated ELISA Kit for TNFα protein content determination	BioGems<br>(Westlake Village, CA, USA)
Bio-Rad protein assay dye reagent concentrate	Bio-Rad Laboratories<br>(Munich, Germany)
2-(4-(2-hydroxyethyl)-1-piperazinyl)-ethanesulfonic acid (HEPES)<br>Acetonitrile<br>Ammonium sulfate ($(NH_4)_2SO_4$)<br>Bromophenol blue<br>Dimethyl sulfoxide (DMSO)<br>Disodium hydrogen phosphate (Na_2HPO_4)<br>Ethylenediaminetetraacetic acid (EDTA)<br>Flavin adenine dinucleotide (FAD)<br>Formaldehyde<br>Glutathione reductase (GR)<br>Glycerin<br>Glycerol<br>Magnesium chloride ($MgCl_2$)<br>NADPH (reduced) tetrasodium salt<br>Nicotinamide adenine dinucleotide phosphate (NADP, oxidized)<br>Phenylmethylsulfonyl fluoride (PMSF)<br>Ponceau S<br>Potassium dihydrogen phosphate (KH_2PO_4)<br>Sodium azide (NaN_3)<br>Sodium chloride (NaCl)<br>Trichloroacetic acid<br>Tris(hydroxymethyl)-aminomethane (Tris)<br>Triton X-100<br>Hydrochloric acid (HCl)	Carl Roth<br>(Karlsruhe, Germany)
deuterium labeled internal and external standards	Cayman Chemical

Primary antibody mouse anti-Prostaglandin E Synthase-1 (microsomal) (mPGES1) (10004350)	(Ann Arbor, MI, USA)
Primary antibody rabbit anti-GPX 1 (3120-1)<br>Primary antibody rabbit anti-NF-κB p65 (D14E12) XP® (8242)<br>Primary antibody rabbit anti-p21WAF1/Cip1 (12D1) (2947)<br>Primary antibody rabbit anti-VIMP (VCP-interacting membrane protein) (D1D1M) (selenoprotein S) (15160S)<br>Secondary antibody horseradish peroxidase (HRP)-coupled goat anti-rabbit IgG (7074S)<br>Secondary antibody HRP-coupled horse anti-mouse IgG (7076S)<br>Secondary antibody anti-mouse IgG Alexa Fluor® 594 Conjugate (8890S)	Cell Signaling<br>(Danvers, MA, USA)
Primary antibody rabbit anti-COX2 (D5H5) XP® (12282)	Epitomics<br>(Burlingam, CA, USA)
Forward and reverse primer for quantitative real-time PCR	Eurofins Genomics<br>(Ebersberg, Germany)
Amersham™ Protran® nitrocellulose membrane (0.2 μm, 300 mm × 4 m)	GE Healthcare<br>(Chicago, IL, USA)
Dithiothreitol (DTT)<br>Protease inhibitor<br>NP-40 Alternative<br>Yttrium	Merck/Millipore<br>(Burlington, MA, USA)
SensiFAST™ cDNA Synthesis Kit for cDNA transcription	Meridian Bioscience<br>(Cincinnati, OH, USA)
PerfeCTa SYBR Green Supermix (2,000 reactions)	Quanta bio<br>(Beverly, MA, USA)
Primary antibody Mouse anti-γ-GCSc (H-5) (GCLC) (sc-390811)	Santa Cruz Biotechnology (Dallas, TX, USA)
3.3′-methylen-bis-(4-hydroxycumarin) (dicumarol)<br>5-sulfosalicylic acid (SSA)<br>β-mercaptoethanol<br>Buthionine-sulfoximine (BSO)<br>D-glucose-6-phosphate<br>Fetal calf serum (FCS)<br>Glucose-6-phosphate dehydrogenase<br>Hydrogen peroxide (H_2O_2)<br>L-Glutathione reduced<br>Lipopolysaccharide (LPS)<br>Menadione<br>Sodium deoxycholate<br>Triphenyltetrazolium chloride (MTT)<br>Trypan blue<br>Tween® 20	Sigma-Aldrich<br>(Steinheim, Germany)
Dynabeads™ mRNA DI-RECT™ Purification Kit for isolation of mRNA<br>GlutaMax<br>Penicillin-streptomycin<br>Roswell Park Memorial Institute 1640 media (RPMI)<br>Selenium (Se) (Honeywell Fluka™)<br>SuperSignal™ West Dura	ThermoFisher Scientific (Waltham, MA, USA)

Trypsin-EDTA solution (1×)	
Phorbol 12-myristate 13-acetate (PMA)	Tocris (Wiesbaden-Nordenstadt, Germany)

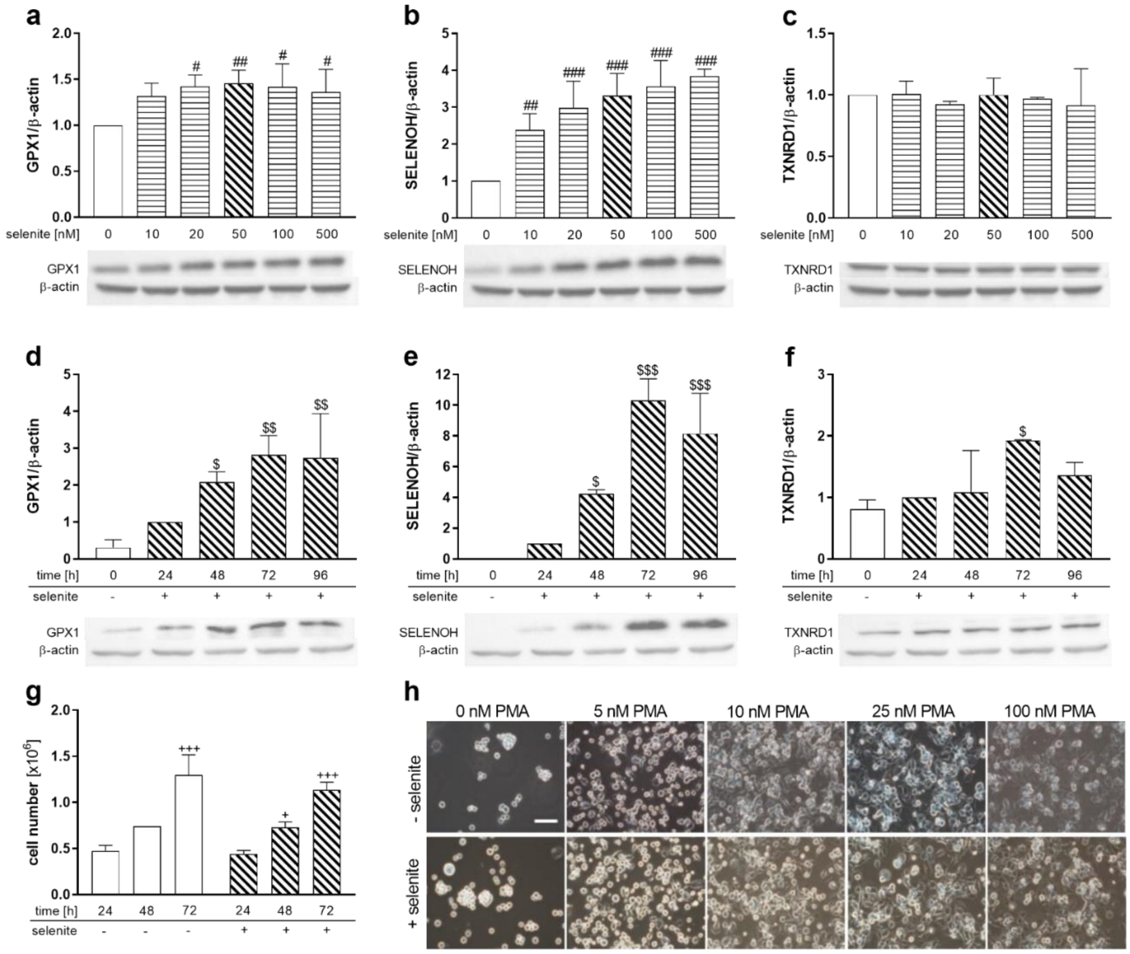

Figure S1. The effect of selenium on the selenoprotein expression in THP-1 monocytes. THP-1 monocytes were treated for 72 h with or without sodium selenite (selenite) at varying concentrations (0-500 nM) **(a-c)** or with or without 50 nM selenite for indicated time (**d-g**). (**a-f**) Protein expression of GPX1 (**a, d**), SELENOH (**b, e**) and TXNRD1 (**c, f**) was to β-actin. - Selenite samples (**a-c**) or – selenite / 24 h samples (**d-f**) were set to 1. Representative blots are shown. (**g**) Cell number of THP-1 monocytes (seeding number 2,5 × 10^5 cells/ml) which were treated with or without 50 nM selenite are given. Results are given as mean + SD (n =3-4). One-way ANOVA (**a-f**) or two-way ANOVA (**g**) with Bonferroni´s post-test. Significant outliers were determined by Grubbs´ test (α = 0.05). $^{\#}$ $P < 0.05$, $^{\#\#}$ $P < 0.01$, $^{\#\#\#}$ $P < 0.001$ vs. cells without selenite (selenite effect); $^{\$}$ $P < 0.05$, $^{\$\$}$ $P < 0.01$, $^{\$\$\$}$ $P < 0.001$ vs. 0 h without selenite; $^{+}$ $P < 0.05$, $^{+++}$ $P < 0.001$ vs. 24 h. (**h**) Representative images of differentiated THP-1 monocytes. The THP-1 cells were pre-treated with or without 50 nM selenite for 72 h and differentiated into macrophages by indicated concentrations of PMA with or without treatment of 50 nM sodium selenite for 48 h. Scale bar, 100 μM.

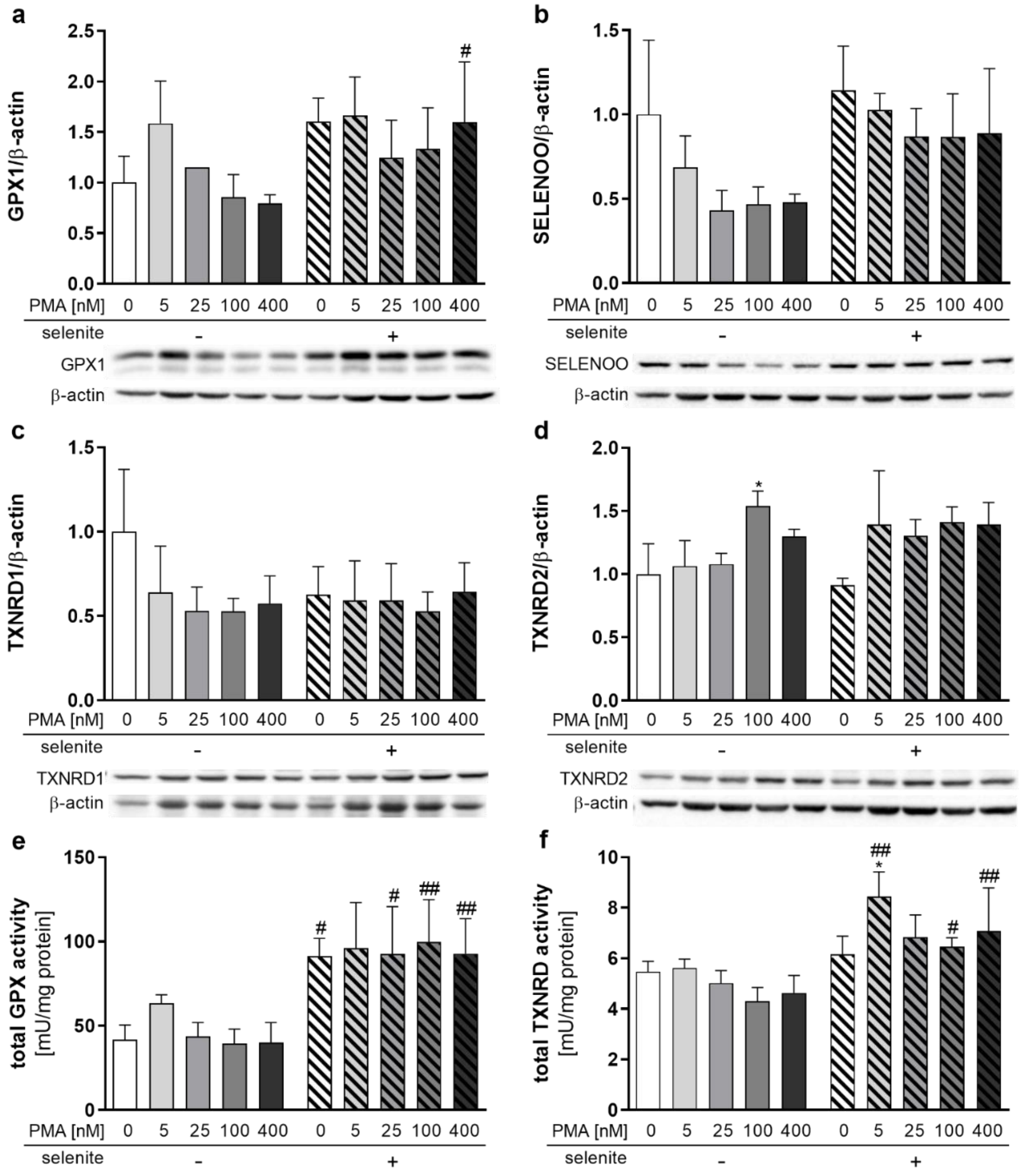

Figure S2. Selenoprotein expression during differentiation. THP-1 monocytes were pre-treated for 72 h with or without 50 nM sodium selenite (selenite) and differentiated into macrophages by treatment with varying PMA concentrations as indicated with or without 50 nM selenite treatment for 48 h. (**a-d**) Protein expression of GPX1 (**a**), SELENOO (**b**), TXNRD1 (**c**), TXNRD2 (**d**) was normalized to β-actin and - PMA/- selenite samples were set to 1. Representative blots are shown. (**e, f**) Total GPX activity (**e**) and total TXNRD activity (**f**). Results are given as mean + SD (n = 3-4). Two-way ANOVA with Bonferroni´s post-test. Significant outliers were determined by Grubbs´ test (α = 0.05). * $P < 0.05$ vs. cells without PMA (PMA effect); $^{\#}$ $P < 0.05$, $^{\#\#}$ $P < 0.01$ vs. cells without selenite (selenite effect).

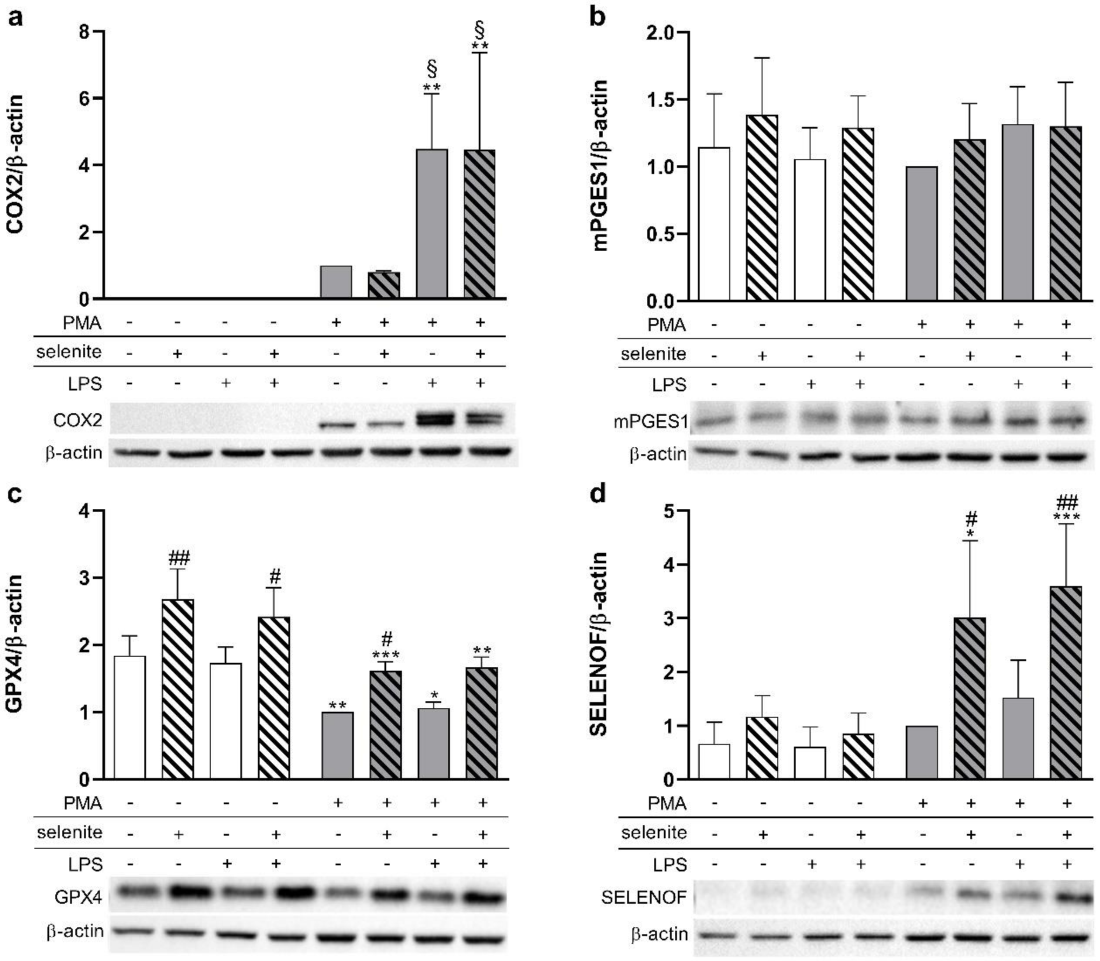

Figure S3. Expression of enzymes involved in lipid mediator biosynthesis and selenoprotein expression. THP-1 monocytes were pre-treated with or without 50 nM sodium selenite (selenite) for 72 h and differentiated into macrophages by 25 nM PMA with or without 50 nM selenite treatment for 48 h and stimulated with 1 μg/ml lipopolysaccharide (LPS) for additional 24 h. (**a**-**d**) Protein expression of COX2 (**a**), mPGES1 (**b**), GPX4 (**c**) and SELENOF (**d**) was normalized to β-actin. + PMA/- selenite/- LPS samples were set to 1. Representative blots are shown. Results are given as mean + SD (n = 4). Three-way ANOVA with Bonferroni´s post-test. Significant outliers were determined by Grubbs´ test ($\alpha = 0.05$). * $P < 0.05$, ** $P < 0.01$, *** $P < 0.001$ vs. cells without PMA (PMA effect); $^{\#}$ $P < 0.05$, $^{\#\#}$ $P < 0.01$ vs. cells without selenite (Se effect); § $P < 0.05$ vs. cells without LPS (LPS effect).
